# Gender Differences in Kinematic Analysis of the Lower Limbs during the Chasse Step in Table Tennis Athletes

**DOI:** 10.3390/healthcare9060703

**Published:** 2021-06-10

**Authors:** Xiaoyi Yang, Yuqi He, Shirui Shao, Julien S. Baker, Bíró István, Yaodong Gu

**Affiliations:** 1 Faculty of Sports Science, Ningbo University, Ningbo 315211, China; yangxiaoyiyiyi@126.com (X.Y.); HeYuqi0809@outlook.com (Y.H.); 2 Centre for Health and Exercise Science Research, Department of Sport, Physical Education and Health, Hong Kong Baptist University, Hong Kong 999077, China; 3 Faculty of Engineering, University of Szeged, 6724 Szeged, Hungary; biro-i@mk.u-szeged.hu

**Keywords:** table tennis, gender differences, chasse step, kinematic

## Abstract

The chasse step is one of the most important footwork maneuvers used in table tennis. The purpose of this study was to investigate the lower limb kinematic differences of table tennis athletes of different genders when using the chasse step. The 3D VICON motion analysis system was used to capture related kinematics data. The main finding of this study was that the step times for male athletes (MA) were shorter in the backward phase (BP) and significantly longer in the forward phase (FP) than for female athletes (FA) during the chasse step. Compared with FA, knee external rotation for MA was larger during the BP. MA showed a smaller knee flexion range of motion (ROM) in the BP and larger knee extension ROM in the FP. Moreover, hip flexion and adduction for MA were significantly greater than for FA. In the FP, the internal rotational velocity of the hip joint was significantly greater. MA showed larger hip internal rotation ROM in the FP but smaller hip external rotation ROM in the BP. The differences between genders can help coaches personalize their training programs and improve the performance of both male and female table tennis athletes.

## 1. Introduction

With increasing global development and interest, table tennis has become popularized all over the world [1]. The positive technical progress of table tennis performance has placed higher requirements on technical and tactical skills and the mental and physical capacity of the players [2]. Technical and tactical skills are considered to be important performance factors in table tennis. These skills include footwork (one-step chasse, slide, cross-step, and pivot) and strokes (forehand, backhand, smash, service, push, top, top counter top, and others) [3]. Footwork is an essential feature of the technical skill portfolio of table tennis athletes, as footwork is the most important prerequisite in the performance of strokes [1]. During training and competition, each stroke changes randomly in response to the opposition. An essential tactical approach to cope with this change is to move using footwork to anticipate the opposition’s shot. Table tennis players need to use footwork to move into a suitable position. Correct footwork allows table tennis players to stroke the ball effectively in the appropriate direction from a strong stance [4]. Therefore, footwork training plays a particularly important role in coaching and performance of the sport and has resulted in much scientific interest.

The lower limb biomechanics of footwork during table tennis performance has attracted much research. Table tennis players usually use three basic footwork patterns, including one-step, side-step, and cross-step, to move into a suitable position to play a forehand topspin shot. Side-step and cross-step movements show higher peak pressure, knee flexion angles, and knee moment than one-step [5]. According to a previous study, the difference in dynamics and kinematics of cross-step movements between professional athletes and novice athletes was that the range of joint motion of professional athletes was obviously smaller and plantar relative load was higher than for novice athletes [6]. We could speculate that the professional athletes had better footwork agility. Furthermore, from analysis of competitions, it has been reported that there are similarities between male and female table tennis athletes in the use of the chasse step [7]. The knee joints in short chasse steps and hip and ankle joints in long chasse steps are more likely to be injured [8]. Yu also compared the chasse step movements of professional athletes with beginners, proving that professional athletes had better foot drive technique; however, the study did not investigate gender differences in order to provide a program for individualized training [9]. Therefore, as the study of footwork becomes more important, gender differences require scientific attention with regards to practice.

Providing opportunities for the development of all elements of table tennis, including footwork, is a crucial part of training and competition. The basic principle of training is individual training [10], the purpose of which is to adjust training programs according to the various individual needs of the table tennis athletes. Athletic diversity stems not only from differences in body structure and athletic skill level, but also from age, gender, or psychological factors [11]. A further variable for consideration is racial differences. Lanzoni et al. compared Asian and European table tennis players and found that Asians used aggressive strokes and footwork more often than European players [4]. There are also differences between levels of table tennis athletes. Elite athletes have higher lower limb drive ability than medium athletes [12]. A recent study compared the topspin forehand loops between different table tennis athletes. The study demonstrated that medium athletes should pay more attention to improving ankle joint ability to reach a higher playing level [13].

There are also differences in male and female table tennis athletes. Gender differences exist in the morphological structure of table tennis players [14]. Zagatto revealed that fat-free mass, fat mass, and body fat percentage values of male table tennis players were higher than female players [15]. Male players are superior than female players in dynamic posture control during multiball table tennis training [16]. According to [17], the male topspin stroke pattern allows for greater use of large muscle groups and joints than for females. Females tend to attack topspin stroke from both sides of the forehand and backhand, while males tend to look for opportunities to hit more powerful topspin stroke from the forehand. These differences may be due to morphological gender variations. By outlining the movement patterns of topspin stroke, differences in male and female contributions to thoracic rotation, external shoulder rotation, dorsal flexion, and supination in the wrist were revealed during the strike stage [18].

Previous studies have described the kinematics of the forehand and backhand upper limbs of table tennis players of different genders. To our knowledge, the kinematics of the lower limbs using a chasse step has not been studied in detail. Therefore, the purpose of this study was to evaluate the differences between genders in the lower limb joint angles, range of motion (ROM), and joint velocity in three planes. Motion time was also processed for further analysis to investigate differences between males and females in table tennis. We hypothesized that male and female table tennis athletes differ in the angle parameters and movement patterns of their lower limb joints during the chasse step. This study may provide practical guidance for table tennis coaches for chasse step training. The findings may also assist in establishing individual training programs for table tennis players to improve and enhance athletic performance. Additionally, the results may also help develop footwork training systems that may prevent injuries.

## 2. Materials and Methods

### 2.1. Participants

As outlined in Table 1, the study involved ten advanced table tennis athletes, five male athletes (MA) and five female athletes (FA), with average ages of 21 ± 2.83 and 21 ± 2.12 y, average body heights of 178 ± 4.24 and 169 ± 4.95 cm, and average body mass values of 74 ± 1.41 and 55 ± 5.66 kg, respectively. All of the participants were members of the table tennis team at Ningbo University, Ningbo, China. All participants were National Division I athletes. All of the participates were right-handed when considering the dominant movement leg of the chasse step. All subjects had no previous lower limb injuries or surgeries for at least six months before the experiment. The offensive style of all participants was the forehand topspin shot, which was used in the study. The subjects were informed about the test procedures and requirements and completed and signed experimentally informed agreements. The study protocol was approved by the Ethics Committee of Research Academy of Grand Health at Ningbo University.

### 2.2. Experimental Setup

Prior to the formal experiment, the height and weight values for all participants were recorded with a calibrated weighing scale and stadiometer. The kinematic information was collected using an 8-camera Vicon motion analysis system (Oxford Metrics Ltd., Oxford, UK) at a frequency of 200 Hz during the test. Vicon Nexus software was used to synchronously capture kinematics data. A total of thirty-six reflective markers (diameter: 14 mm) were used (these included anterior and posterior (left and right) superior iliac spine; medial and lateral (left and right) condyle; medial and lateral (left and right) malleolus; first and fifth (left and right) metatarsal heads, distal interphalangeal joint (left and right) of the second toe, as well as a total of six tracking clusters attached to the middle and lateral (left and right) thigh, shank, and heel [19,20]. Previous studies have demonstrated that footwork patterns are very reliable for both intra and inter-observations using current classifications and methods.

**Figure 1:**
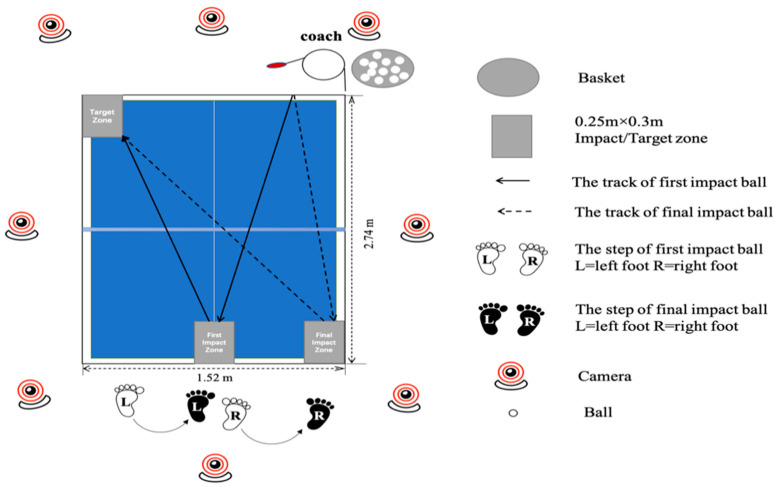
Experimental set up including the backward phase (BP) and forward phase (FP) during the chasse step.

As outlined in Figure 1, the test was conducted at the Table Tennis Training Center at Ningbo University. The experimental process included one target zone and two impact zones (0.25 m × 0.3 m) traced upon a professional table tennis table (Rainbow, Double Happiness Sports Company, Shanghai, China).

All participants were informed of the experimental requirements and procedures. Before data collection, all participants had time to become familiar with the experimental environment. They were required to warm up for 20 min and to perform a multiball training regime for 10 min that incorporated the chasse step. Participants hit the ball (D40+, Double Happiness Sports Company, Shanghai, China) using a forehand topspin during the warm-up and test. During the test, all the participants wore the same table tennis match shoes and used the same table tennis racket. Participants were asked to hit balls with their maximum effort onto the target zone (diagonal side), just as they would in a formal match. Each participant successfully completed at least ten performances and the kinematics of each test were recorded synchronously.

### 2.3. Definition of the Motion Phase

**Figure 2:**
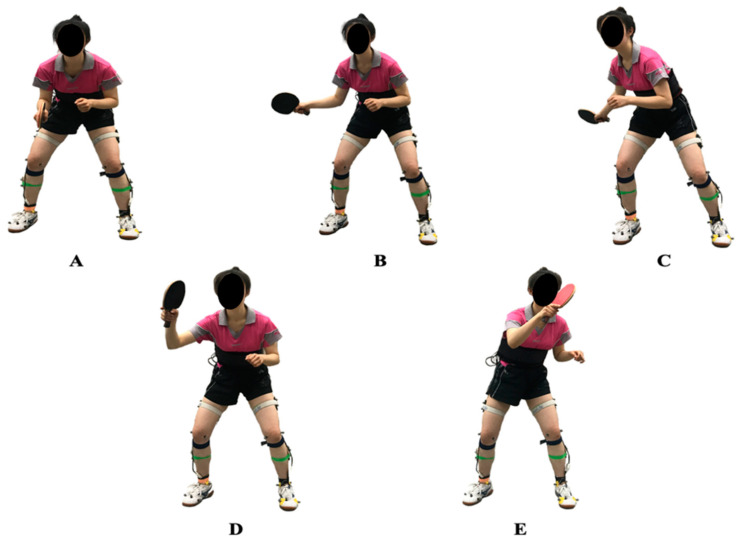
The division and definition of the motion phase. The motion phase (**A**) was defined as the natural position (NP). (**B**,**C**) The backward swing (BS) phase during the chasse step. (**D**,**E**) The forward swing (FS) phase during the chasse step. This research focused on the key event of the entire motion cycle of the chasse step, so we defined position C as the event that defined the end moment of the backward phase (BP). Position E was defined as the key event that identified the end moment of the forward phase (FP).

As outlined in Figure 2, motion phase A was defined as a natural position (NP). Figure 2B,C shows the backward swing (BS) phase during the chasse step. Figure 2D,E shows the forward swing (FS) phase during the chasse step. This study focused on the key events of the entire motion cycle of the chasse step, so we defined position C as the key event, which meant the end moment of the backward phase (BP). Position E was defined as the key event that signified the end moment of the forward phase (FP).

### 2.4. Data Processing

The chasse step refers to two specific movements, namely the backward phase (BP) and forward phase (FP) of the dominant foot in a footwork movement. The data for the lower limb joint angles, range of motion (ROM), joint velocity, and motion time in the sagittal, frontal, and transverse planes during the entire motion cycle for each participant were recorded for further processing and analysis. 

### 2.5. Statistical Analysis 

Data were analyzed using SPSS (Version 20.0, Chicago, IL, USA) and the statistical significance level was set to 0.05. The Shapiro–Wilk test was used to verify the normal distribution of variables. Independent t-tests were performed to determine the kinematic differences between the chasse step movements of male and female table tennis players for each variable of interest. The analysis included joint angle, motion time, joint velocity, and range of motion (ROM) measurements of the ankle, knee, and hip joints. MATLAB scripts were written to extract joint angle, ROM, and joint velocity values and enabled calculation of the differences.

Data were exported to MATLAB R2017A (The MathWorks, Natick, MA, USA) and processed using scripts written for specific data sets. The open-source SPM 1D script (independent t-test, significance threshold of 0.05) was used for statistical analysis. The chasse step SPM 1D analysis was performed using a custom MATLAB script [21] and a separate curve was generated for each task before the analysis. All extracted data were extended to time series curves of 101 data points, representing the entire motion cycle of the chasse step [22]. 

## 3. Results

### 3.1. Motion Time

As shown in Table 2, the times for the entire motion cycles for MA and FA in the chasse step were 1.01 ± 0.03 s and 1.01 ± 0.04 s, respectively. The time for MA was significantly shorter than that for FA in the BP but longer in the FP. Moreover, there was a significant difference in the times between FA and MA throughout the entire motion cycle.

### 3.2. Lower Limb Joint Angle

**Figure 3:**
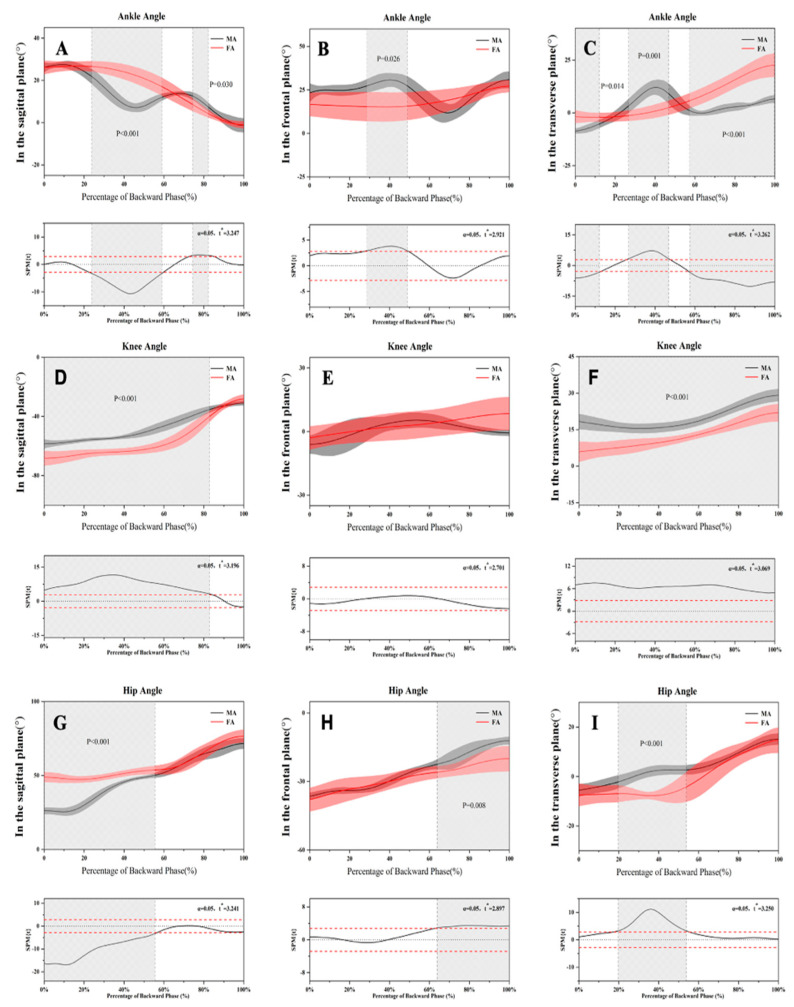
Changes of the lower limb joint angles during the backward phase in three planes. (**A**–**C**) Ankle angle changes during the BP in three planes. (**D**–**F**) Knee angle changes during the BP in three planes. (**G**–**I**) Hip angle changes during the BP in three planes.

**Figure 4:**
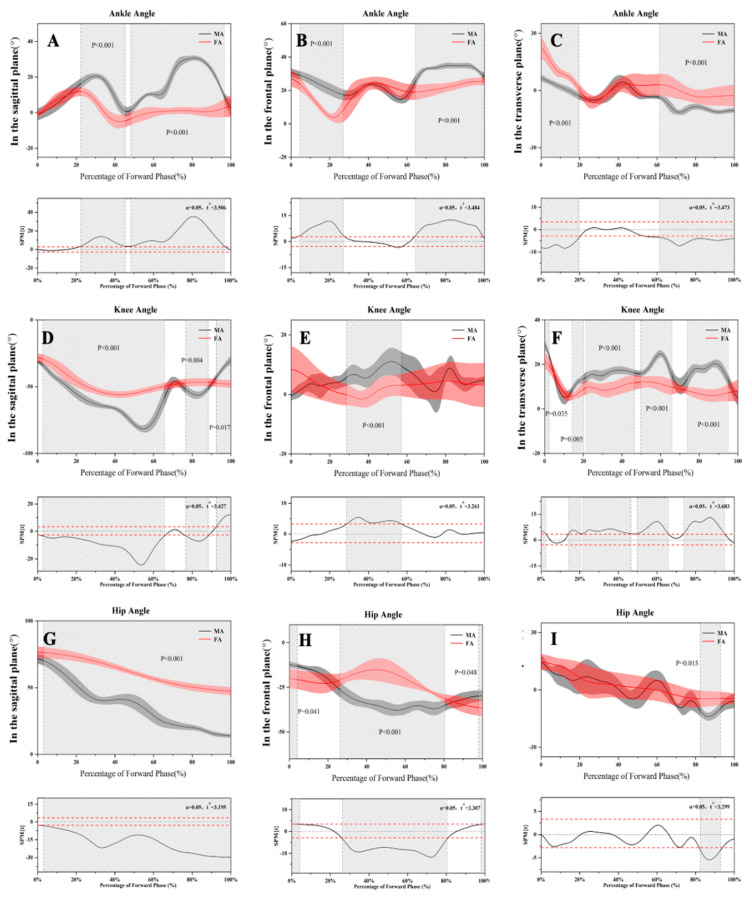
Changes of the lower limb joint angles during the forward phase in three planes. (**A**–**C**) Ankle angle changes during the FP in three planes. (**D**–**F**) Knee angle changes during the FP in three planes. (**G**–**I**) Hip angle changes during the FP in three planes.

Table 3 shows the joint angles for the BP and FP in the sagittal, frontal, and transverse planes for both MA and FA. Figure 3 and Figure 4 show the kinematic differences for the BP and FP of the chasse step for male and female athletes.

In the sagittal plane, MA showed significantly less knee flexion in the BP as compared to FA but significantly greater knee extension in the FP during the whole procedure of the chasse step. The hip flexion for MA was significantly lower than that for FA over the entire motion cycle. In the frontal plane, knee abduction for MA was significantly lower in the BP than for FA. The hip joint adduction and abduction for MA were significantly greater than for FA in the BP and FP, respectively. In addition, FA had significantly greater internal and external rotation than MA in the transverse plane in the BP and FP. However, the knee joints of MA showed greater external rotation than for FA during the BP.

### 3.3. Range of Motion

**Figure 5:**
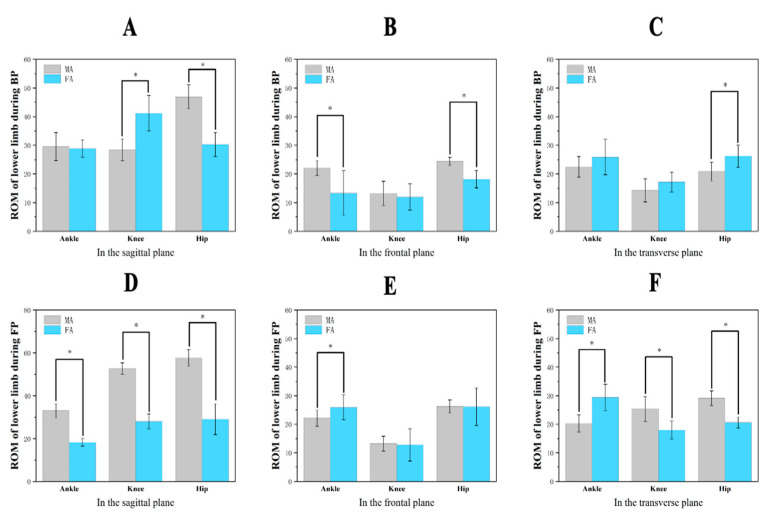
Comparison of ROM values in the BP and FP between MA and FA. (**A**–**C**) Lower limb ROM changes during the BP in three planes. (**D**–**F**) Lower limb ROM changes during the FP in three planes. * Significant differences at the hip, knee, and ankle (*p* < 0.05).

The ROM values for MA and FA in all planes during BP and FP are shown in Table 4 and Figure 5. The lower limb ROM values for MA and FA were significantly different in the BP and FP. In the sagittal plane, MA significantly increased their ROM in ankle flexion compared with FA in the FP. The knee flexion was significantly lower in the BP than for FA but the knee extension was significantly greater in the FP. The hip flexion for MA was significantly greater than that of FA throughout the entire motion cycle. In the frontal plane, MA had significantly larger inversion in the BP and less eversion in the FP than FA. During the BP, MA showed significantly greater hip abduction than FA. In the transverse plane, MA exhibited less external rotation of the ankle and greater internal rotation of the knee than FA during the FP. Compared with FA, MA showed less external hip rotation in the BP and greater internal hip rotation in the FP.

### 3.4. Lower Limb Joint Velocity

**Figure 6:**
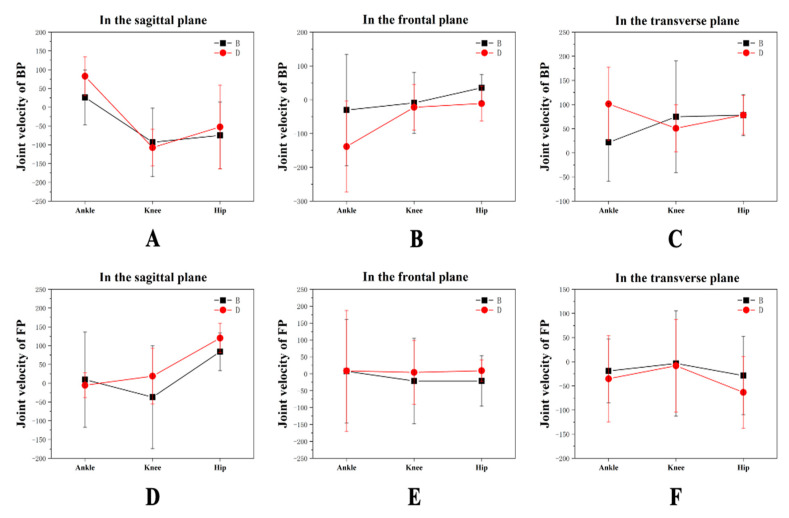
Comparison of the joint velocity values in the BP and FP between MA and FA. (**A**–**C**) Joint velocity values for the lower limb during the BP in three planes. (**D**–**F**) Joint velocity values for the lower limb during the FP in three planes.

Table 5 and Figure 6 show the joint velocity values in the BP and FP in all planes for MA and FA. The velocity of the ankle in the sagittal plane was significantly lower for MA than for FA. In both the BP and FP, the velocity values for the knee joint and hip joint for MA were also significantly lower than for FA. However, in the frontal plane, MA showed significantly greater velocity values for the ankle and hip joints than for FA in the BP. The hip velocity for MA was lower than for FA in the FP. In the transverse plane, the velocity for the ankle joint for MA in the BP was significantly lower than for FA. There were no significant differences between the knee joint values in the BP and FP. For MA, the velocity value for the hip joint was significantly greater in the FP.

## 4. Discussion

The objective of this research was to analyze gender differences based on the lower limb kinematics of the table tennis chasse step. The key findings of this study were as follows. In terms of time spent, there was a significant difference between MA and FA for the entire motion cycle. Moreover, there were significant differences between male and female table tennis athletes in the backward phase (BP) and forward phase (FP). 

Hip flexion for MA was significantly lower than for FA, while hip adduction and abduction were significantly greater during the entire motion cycle. The knee abduction for MA was significantly lower than for FA in the BP and MA showed greater external rotation than FA. Compared to FA, MA showed less knee flexion in the BP but greater knee extension in the FP. The hip flexion ROM for MA was significantly greater than for FA throughout the entire motion cycle. MA showed significantly greater hip abduction (ROM) than FA in the BP. Compared with FA, MA showed less hip external rotation (ROM) during the BP and greater hip internal rotation (ROM) during the FP. For MA, the internal rotational velocity of the hip joint was significantly greater in the FP.

In terms of time, MA had quicker times in the BP but longer times in the FP than FA during the chasse step. The entire motion cycle took about one second for both MA and FA, which indicates that male table tennis athletes have a longer forward phase. This could demonstrate that male athletes could be prepared for short periods of intense activity during the chasse step. This is consistent with previous research. Table tennis players have limited time to hit the ball, so it is beneficial for them to improve their swing speed in a short time [23]. Table tennis players prepare for quick shots, which requires physical control. However, the dynamic posture control ability of female players is less than that of male table tennis players [16]. This may be related to the body compositions of males and females. Body composition is a very important parameter of athletic performance. Male table tennis athletes have higher muscle mass than females. Högström et al. observed similar results. They studied male and female teenage adolescent cross-country and alpine skiers. Their results were consistent with research on adult cross-country skiers, where adult males generally exhibit higher muscle mass [24]. We could suggest that male athletes have better body control skills and stronger lower limb muscles to stabilize their bodies during the chasse step.

During the BP stage, MA exhibited greater inversion and internal rotation of the ankle and greater joint velocity during inversion of the ankle compared to FA. Moreover, knee flexion was significantly lower in the BP but extension was significantly greater in the FP than for FA. The external and internal rotation for MA in the BP and FP were both greater than for FA. MA have a greater range of joint movement. This may mean that MA are better prepared for the lower limb than FA during BP. High-level athletes can effectively use the knee joint to rotate the upper limbs, which can result in more body movements than low-level athletes [25]. Muscle tissue around the knee joint is crucial in maintaining the stability of the joint. The ability of the knee to maintain stability under rapidly changing loads is an important factor affecting the dynamic stability of the knee [26]. Overuse of the knee will cause overload injuries, especially of the ACL [27]. Therefore, we speculate that the risk of ACL injury for female athletes is lower than that for male athletes. During chasse step training for table tennis athletes, male table tennis players should pay attention to improving the strength of the knee joint, enhancing the stability of the knee, and reducing the possibility of knee joint injury. 

A further finding was that hip flexion and adduction were significantly greater for MA than for FA. MA showed larger hip internal rotation ROM in the FP but smaller hip external rotation ROM in the BP. Moreover, the internal rotational velocity of the hip joint was significantly greater in the FP. In racket sports, one of the most critical factors is the rotation of the trunk axis [28]. The speed of the racket is not only determined by the playing proficiency of the athlete but also by the involvement of the torso. The core is a very important part of the torso. The core includes the lumbopelvic–hip joint complex and its surrounding muscle tissue. It provides dynamic stability for the entire dynamic chain during the process of functional movement [29,30]. In the process of movement, hip movement is indispensable from the role of the core. Hip movement is an important factor for energy generation and energy transfer in the performance of table tennis [12]. The peak joint angle in hip flexion can increase the speed of the ball [8]. It could be suggested that male table tennis players are more likely to turn their bodies when hitting the ball than female players. Lanzoni also found that male play is dominated by the pivot, while female play is dominated by the slide step [7]. Males have faster hip rotation to generate more energy during the backward phase than females and males have faster hitting speeds; therefore, male table tennis players may have greater core and hip muscle strength than female players. During the training of the table tennis chasse step, it is important to strengthen the core muscles and improve ball speed. These factors are very important for athletes (especially female athletes) and should improve their sports performance.

The results of this study could be useful for table tennis coaches and athletes. The variation of movement parameters between individuals reflects that the sports skills of male and female table tennis athletes are very individualized. Therefore, coaches should pay attention to gender differences between table tennis players when creating or adopting techniques and tactics for athletes during chasse step training. For table tennis players, individualized training programs require consideration to prevent sports injuries and improve sports performance. The coordination of the movement patterns of the chasse step can also be considered as important practical outcomes of this study.

There are several limitations that need consideration in this study. Firstly, we only looked at male and female table tennis players at the same level. It would also be useful to study table tennis players of different training levels and different ages. Secondly, this study only compared the biomechanical differences of the dominant lower extremity. Non-dominant limbs may also contribute significantly to athletic performance. Thirdly, only the kinematics data for the chasse step and the kinetics data were collected. In future studies, different subject populations should be considered, investigating the relationships between the upper and lower limbs and the combined biomechanical characteristics in combination with use of the racket.

## 5. Conclusions

In this study, we systematically analyzed the lower limb kinematics of advanced table tennis athletes of different genders using the chasse step. We outlined the lower limb joint movements of advanced table tennis athletes of different genders and the use of the chasse step. The findings from this study suggest that coaches and athletes when practicing the chasse step should pay attention to the role of the knee joint. Attention should be focused on strengthening the knee and core to reduce the risk of injury during the chasse step. The differences observed between genders in this study can provide a reference for coaches to personalize and customize programs during training, resulting in improved competition performance in table tennis athletes. It can also give guidance and suggestions for the training of male and female athletes in other sports.

## Figures and Tables

**Table 1:** The participant information (mean ± SD).

Population	Gender	Age (Year)	Height (cm)	Weight (kg)	Experience (Year)
5	male	21 ± 2.83	178 ± 4.24	74 ± 1.41	14 ± 2.12
5	female	21 ± 2.12	169 ± 4.95	55 ± 5.66	12 ± 2.12

**Table 2:** Comparison of means ± standard deviations for BP and FP times between MA and FA (unit: seconds).

Variables	MA	FA	*p*
Backward phase (BP)	0.27 ± 0.03	0.48 ± 0.05	0.000 *
Forward phase (FP)	0.74 ± 0.03	0.53 ± 0.05	0.000 *
Entire motion cycle	1.01 ± 0.03	1.01 ± 0.04	0.040 *

Note: * Significant differences between MA and FA (*p* < 0.05). BP, backward phase; FP, forward phase; MA, male athletes; FA, female athletes.

**Table 3:** Comparison of means ± standard deviations for joints angles during key events of the BP and FP between MA and FA (unit: degrees).

Variables		BP			FP		
		MA	FA	*p*	MA	FA	*p*
**Ankle**	X	−1.31 ± 3.56	−1.10 ± 1.86	0.871	1.64 ± 5.51	3.88 ± 6.59	0.420
	Y	30.89 ± 2.87	27.22 ± 5.30	0.075	27.60 ± 3.11	25.68 ± 2.65	0.155
	Z	6.58 ± 1.99 *	22.65 ± 5.98 *	0.000	−10.53 ± 1.23 *	−2.66 ± 6.07 *	0.003
**Knee**	X	−30.84 ± 1.79 *	−28.05 ± 3.20 *	0.027	−29.65 ± 3.80 *	−48.09 ± 3.09 *	0.000
	Y	−0.65 ± 0.92 *	8.49 ± 6.82 *	0.037	4.80 ± 0.74	3.16 ± 4.23	0.654
	Z	29.23 ± 2.72 *	21.95 ± 3.80 *	0.000	3.99 ± 3.73	8.17 ± 5.29	0.058
**Hip**	X	71.74 ± 3.82 *	76.59 ± 4.61 *	0.020	13.96 ± 1.43 *	47.39 ± 3.29 *	0.000
	Y	12.05 ± 1.05 *	−20.04 ± 7.32 *	0.007	−29.57 ± 1.80 *	−36.61 ± 5.96 *	0.005
	Z	15.18 ± 2.44	14.73 ± 5.41	0.818	−5.75 ± 2.12	−4.62 ± 3.56	0.401

Note: * Significant differences at the hip, knee, and ankle (*p* < 0.05); X, the sagittal plane; Y, the frontal plane; Z, the transverse plane. BP, backward phase; FP, forward phase; MA, male athletes; FA, female athletes.

**Table 4:** Comparison of means ± standard deviations for ROM values in the BP and FP between MA and FA (unit: degrees).

Variables		BP			FP		
		MA	FA	*p*	MA	FA	*p*
**Ankle**	X	29.5 ± 4.90	28.89 ± 3.02	0.742	33.04 ± 3.26 *	18.23 ± 1.88 *	0.000
	Y	22.16 ± 2.61 *	13.34 ± 7.81 *	0.006	22.15 ± 2.78 *	25.97 ± 4.43 *	0.033
	Z	22.51 ± −3.59	25.90 ± 6.14	0.153	20.37 ± 2.99 *	29.43 ± 4.57 *	0.000
**Knee**	X	28.39 ± 3.84 *	41.15 ± 6.22 *	0.000	52.68 ± 2.76 *	27.98 ± 3.56 *	0.000
	Y	13.19 ± 4.23	11.97 ± 4.57	0.542	13.25 ± 2.60	12.81 ± 5.68	0.827
	Z	14.30 ± 4.02	17.19 ± 3.46	0.102	25.42 ± 4.35 *	18.00 ± 3.17 *	0.000
**Hip**	X	46.89 ± 4.20 *	30.30 ± 4.16 *	0.000	57.58 ± 3.92 *	29.07 ± 7.21 *	0.000
	Y	24.44 ± 1.41 *	18.17 ± 3.00 *	0.000	26.29 ± 2.23	26.18 ± 6.54	0.972
	Z	20.86 ± 3.28 *	26.22 ± 3.82 *	0.003	29.15 ± 2.54 *	20.66 ± 1.94 *	0.000

Note: * Significant differences at the hip, knee, and ankle (*p* < 0.05); X, the sagittal plane; Y, the frontal plane; Z, the transverse plane. BP, backward phase; FP, forward phase; MA, male athletes; FA, female athletes.

**Table 5:** Comparison of means ± standard deviations for the joint velocity values in the BP and FP between MA and FA (unit: degrees/second).

Variables		BP			FP		
		MA	FA	*p*	MA	FA	*p*
**Ankle**	X	26.24 ± 73.20 *	82.49 ± 51.92 *	0.000	9.47 ± 126.75	−5.53 ± 33.29	0.252
	Y	−30.51 ± 164.76 *	−138.47 ± 134.44 *	0.000	7.60 ± 153.50	8.61 ± 178.52	0.966
	Z	21.66 ± 80.72 *	101.37 ± 75.85 *	0.000	−19.16 ± 66.08	−35.29 ± 89.32	0.146
**Knee**	X	−93.27 ± 90.92	−107.48 ± 49.04	0.169	−37.38 ± 136.87 *	18.75 ± 74.07 *	0.000
	Y	−9.43 ± 90.12	−22.26 ± 67.45	0.254	−21.42 ± 126.28	4.09 ± 94.50	0.106
	Z	74.75 ± 115.60	50.85 ± 48.91	0.058	−3.51 ± 109.19	−8.39 ± 95.80	0.736
**Hip**	X	−74.89 ± 88.77	−52.43 ± 111.27	0.114	83.64 ± 50.28 *	120.03 ± 38.97 *	0.000
	Y	35.18 ± 39.47 *	−11.19 ± 51.75 *	0.000	−21.22 ± 74.46 *	9.17 ± 31.86 *	0.000
	Z	78.07 ± 42.48	78.40 ± 40.32	0.956	−28.68 ± 81.02 *	−63.54 ± 74.13 *	0.002

Note: * Significant differences at the hip, knee, and ankle (respectively) (*p* < 0.05); X, the sagittal plane; Y, the frontal plane; Z, the transverse plane. BP, backward phase; FP, forward phase; MA, male athletes; FA, female athletes.

## Data Availability

The data that support the findings of this study are available on reasonable request from the corresponding author. The data are not publicly available due to privacy or ethical restrictions.
